# Stable Thalamic Topography Across Perinatal Development: a Diffusion MRI Study

**DOI:** 10.1007/s10548-026-01257-9

**Published:** 2026-10-12

**Authors:** Hui Ji, Zhexin Wu, Anna Speckert, Ruth Tuura, Cornelia Hagmann, Walter Knirsch, Andras Jakab

**Affiliations:** 1 Center for MR Research, University Children’s Hospital Zurich, Lenggstrasse 30, Zurich, CH-8008 Switzerland; 2 Neuroscience Center Zurich, University of Zurich, Zurich, Switzerland; 3 Children’s Research Center, University Children’s Hospital Zurich, Zurich, Switzerland; 4 Department of Information Technology and Electrical Engineering, ETH Zurich, Zurich, Switzerland; 5 University Research Priority Program (URPP), Adaptive Brain Circuits in Development and Learning (AdaBD), University of Zurich, Zurich, Switzerland; 6 Department of Neonatology, University Children’s Hospital Zurich, Zurich, Switzerland; 7 Pediatric Cardiology, Pediatric Heart Center, Department of Surgery, University Children’s Hospital Zurich, Zurich, Switzerland

**Keywords:** Diffusion MRI, Thalamus, Development, Newborns, Preterm birth

## Abstract

The thalamus is critical relay hub of brain connectivity, yet its topographical organization during the early human brain development remains incompletely understood. Our study characterized the critical perinatal developmental period by optimizing and applying a clustering framework to parcellate the neonatal thalamus and delineate its developmental trajectory.

Thalamus topography was studied using Gaussian Mixture Model (GMM) based clustering applied to voxel-level fiber orientation distribution functions, obtained from diffusion MRI data. Three perinatal datasets were used: term-born infants from developing human connectome project (dHCP, *n* = 68, age at MRI: 37.4–44.7 weeks), dHCP preterm infant data (*n* = 48, 29.3–37.4 weeks) and a neonatal control dataset from Zürich (*n* = 42, 39.0-48.7 weeks). Clustering was optimized using an adult test-retest diffusion MRI dataset (*n* = 30).

We subdivided the thalamus into seven clusters, where GMM showed moderate reproducibility (test-retest overlap ranged from 0.65 to 0.73), and comparable anatomical alignment with digital Morel atlas. Robust regression analysis revealed significant age-related volume increases in absolute volumes across thalamic clusters (FDR-corrected *p* < 0.001). Topography by means of relative volumes remained stable, suggesting preservation of thalamic topography during late gestation. Two clusters, closely corresponding to the ventral-anterior nucleus and pulvinar group, showed moderate age-related topographical changes (FDR-corrected *p* < 0.05, adjusted R²=0.039 and 0.101). Group-wise analysis of harmonized volumes revealed no significant group effects between preterm and term born neonates. No sex differences were observed.

Our findings demonstrate that thalamic topography is largely established across the perinatal period; however, the lack of systematic term-equivalent scans in preterm infants limits our ability to draw conclusions about the effects of prematurity. The interpretation might be limited by the indirect nature of diffusion MRI features and the moderate reproducibility of the clustering techniques.

**Supplementary Information:**

The online version contains supplementary material available at https://doi.org/10.1007/s10548-026-01257-9.

## Introduction

The thalamus is essential for neural circuit development, driving key processes such as axonal guidance and neuronal migration (Miller et al. 1993; Auladell et al. 2000; Sur and Rubenstein 2005; Jones 2007). It plays an important role not only as a sensory relay but also by modulating information exchange between cortical regions, supporting sensorimotor, cognitive, emotional, and attentional functions. Disruptions in thalamic development have been linked to various developmental or psychiatric conditions, including bipolar, autism, cognitive impairment, and emotional dysfunctions (Carrera and Bogousslavsky 2006; Kubota et al. 2013; Nair et al. 2013; Alcauter et al. 2014; Anticevic et al. 2014; Nosarti et al. 2014). Preterm birth has also been associated with altered thalamic development and organization (Ball et al. 2012, 2013, 2015).

During early brain development, the thalamus establishes a topological structure that forms the scaffolding for neural pathways and reciprocal connection with cortex, significantly influencing cortical development. Unique aspects of human brain development, such as the abundance of prefrontal connections and neuron counts in the mediodorsal nucleus (Abitz et al. 2007), make it difficult to extrapolate findings from other species, and our understanding of how pathologies might affect thalamus development needs further research on human data. Thalamic circuitry development in neonates is particularly sensitive. Preterm infants were found to show reduced thalamic volume, which reflects a possible impact of premature birth on thalamic maturation and overall brain architecture (Ball et al. 2012; Nosarti et al. 2014; Alexander et al. 2019; Thompson et al. 2020; Menegaux et al. 2021).

Thalamic nuclei are traditionally delineated using histological methods and manual segmentation. However, advancements in MRI, particularly diffusion MRI (dMRI), functional MRI (fMRI) have enabled in vivo parcellation, that is, the data-driven subdivision of the thalamus structure into sub-compartments sharing certain properties derived from MRI quantification. This technique revealing detailed anatomy and connectivity and incorporating patient-specific information (Iglesias et al. 2018; Su et al. 2019). fMRI based thalamus mapping shows functional specialization, but it was not found to be well aligned with the histological atlases (Iglehart et al. 2020). DTI has become a widely used approach for thalamic parcellation, and has been used to reveal both global (that is, thalamo-cortical) or more localized fiber connectivity (Wiegell et al. 2003; Ziyan et al. 2006; Jonasson et al. 2007; Jbabdi et al. 2009; Yamada et al. 2010; Ye et al. 2013).

Thalamus parcellation can be performed using long-range as well as local properties, such as the main orientation of white matter axonal bundles within a voxel (Behrens et al. 2003; Kumar et al. 2015; Battistella et al. 2017). However, in the developing human brain, pre-myelinated axons reduce diffusion anisotropy diminish the accuracy of long-range connectivity predictions (Dubois et al. 2014). While DTI models dMRI signal at a local scale, based on diffusion orientations, can already identify thalamic subdivisions but may produce spatially disconnected regions due to its focus on main fiber directions only (Unrath et al. 2008; Mang et al. 2012; Kumar et al. 2015). A conceptually different approach is to incorporate information beyond the principal orientation of white matter diffusion, which improved clustering accuracy (Battistella et al. 2017). The constrained spherical deconvolution (CSD) approach, especially the single-shell 3-tissue variant (SS3T-CSD), estimates white matter fiber orientation distributions (FODs) while explicitly modeling grey matter and cerebrospinal fluid (CSF). This makes it likely more suited for the neonatal brain, which has high free water content (Dhollander and Connelly 2016; Dhollander et al. 2019).

The perinatal and early postnatal period is characterised by a rapid increase of sensory stimuli to the brain, which affects thalamo-cortical connectivity or thalamic structure development in general. Since we lack a complete understanding of how thalamus topography might change in this developmental period, our study aimed to describe the developmental trajectory of thalamic topographical organization by using local diffusion properties as a proxy to thalamus connectivity and structure. Building on previous work validating the use of local diffusion features for thalamic parcellation, we hypothesize that data-driven clustering of the thalamus into sub-compartments based on the white matter FODs sufficiently reflect thalamic topography in early postnatal development. We further aimed to optimize clustering techniques to generate anatomically meaningful subdivisions.

## Methods

The first part of our study focused on developing and optimizing a thalamus clustering pipeline using FODs derived from DTI. We employed a scan-rescan dataset of healthy adults for this purpose (Boekel et al. 2016). An adult dataset was chosen due to the availability of histological atlases, high scan quality, and scan-rescan data. In "Features for Clustering" section and "Bayesian Hyperparameter Tuning" section, we performed feature selection and clustering parameter tuning using grid search and Bayesian optimization on this adult dataset. Clustering accuracy was evaluated in "Evaluation of Clustering Accuracy" section by computing both scan-rescan Dice similarity and Dice overlap with digital Morel atlas. After parameters optimization, we applied Gaussian Mixture Models (GMM) clustering to developmental MRI data taken from the Developing Human Connectome Project (dHCP), which includes both preterm and term-born infants, and a dataset comprising neonates scanned in Zurich. These later were used to assess thalamic growth trajectories, evaluate scan-related variability, and assess the effects of preterm birth on thalamic volume and topography. The code for clustering is publicly available at https://github.com/10258392511/Clustering.

### Study Population and MRI Acquisition

#### Scan-rescan Dataset: Demographics and MRI Acquisition

This dataset consists of healthy adult subjects, previously described by Boekel et al. (Boekel et al. 2016), and retrieved from the test-retest repository (http://www.nitrc.org/projects/dwi_test-retest/). A total of 34 healthy young subjects underwent two MRI scans on the same day using a 3T Philips Achieva XT scanner with a 32-channel head coil. Due to post-processing issues, four subjects were excluded: one for slice thickness discrepancy between scans, one for poor T1-weighted MRI to dMRI registration, and two for errors in registering the thalamus atlas to individual thalamic structures. The final cohort included 30 participants.

T1-weighted scans were acquired using a turbo field echo sequence with the following parameters: TR/TE = 8.4/3.9 ms, flip angle = 8°, voxel size = 1 × 1 × 1 mm^3^. Diffusion MRI was conducted in two sessions, each consisting of four repeated scans. A multi-slice spin echo, single-shot DWI sequence was used with TR/TE = 7.545/86 ms. voxel size = 2 × 2 × 2 mm^3^, 60 transverse slices, and diffusion weighting along 32 gradient directions (b = 1000 s/mm^2^). Six non-diffusion weighted images (b0; b = 0 s/mm^2^) were also acquired and averaged. To minimize motion artifacts, only the first diffusion MRI scan from each session was used in the subsequent analysis.

#### Scan-rescan Dataset: MRI Processing

The thalamus proper (= thalamus outlines excluding the LGN and MGN) was manually annotated on 3D T1 images using 3D Slicer (Kikinis et al. 2014), version 4.8. To transform the structural thalamus mask to diffusion space, we followed these steps: First, we extracted the *b* = 1000 s/mm^2^ images from the diffusion MRI data and calculated their median using *dwiextract* in MRtrix3 (Tournier et al. 2019) and *fslmaths*. Bias field correction was then applied to the median *b* = 1000 s/mm^2^ image using *dwibiasfield* in ANTs (Tustison et al. 2010). Next, the 3DT1 images were aligned to the bias-corrected median b = 1000 s/mm^2^ images via linear registration using FLIRT in FSL with 6 degrees of freedom (DOF) (Jenkinson and Smith 2001). The linear transformation was converted to ANTs compatible format using the c3d_affine_tool from the c3d software. We then performed linear and non-linear registration of the 3D T1 images to the median b = 1000 s/mm² image using antsRegistrationSyN in ANTs (Avants et al. 2008), initializing the process with the previously estimated linear transformation matrix. Finally, the thalamus masks were transformed from 3DT1 space to diffusion space using *antsApplyTransform* (Avants et al. 2008) and threshold at 0.5 in diffusion space.

#### Developing Human Connectome Project Data: Demographics and MRI Acquisition

This dataset was used to examine thalamic topography development in normal newborns and prematurely infants. This data was sampled from the open-source Developing Human Connectome Project (dHCP) neonatal release 2 (Bastiani et al. 2018), accessed in January 2022. We sampled subjects to achieve an approximately equal number of cases per gestational week at the time of scan, including both preterm and term-born infants. The sampled dataset included MRI and demographic data (birth age, scan age, sex) for 120 subjects. After excluding four cases due to unavailable diffusion MRI data, a total of 116 cases (56 females, 60 males) were analyzed. The birth ages ranged from 25.6 to 42 weeks (mean ± SD: 36.8 ± 4.3), with scan ages ranging from 29.3 to 44.7 weeks (mean ± SD: 38.5 ± 4.02). The cohort included 48 preterm infants with birth ages between 25.6 and 36.9 weeks (mean ± SD: 32.5 ± 3.1) and scan ages between 29.3 and 37.4 weeks (mean ± SD: 34.4 ± 2.02). Additionally, 68 term-born infants were included, with birth ages from 37 to 41.9 weeks (mean ± SD: 39.9 ± 1.3) and scan age ranging from 37.4 to 44.7 weeks (mean ± SD: 41.4 ± 2.1). Because preterm infants were not systematically scanned at term-equivalent age, cohort comparisons were interpreted as exploratory and not as accurate estimates of prematurity-related effects at comparable developmental stages.

Anatomical MRI: T2-weighted images were acquired with a Turbo Spin Echo (TSE) sequence and processed with super-resolution reconstruction (Kuklisova-Murgasova et al. 2012) and motion correction (Cordero-Grande et al. 2018) to obtain a 3D volume (referred to as 3DT2) with an isotropic resolution of 0.5 × 0.5 × 0.5 mm³. Detailed MRI acquisition parameters were provided in Makropoulos et al. (Makropoulos et al. 2018).

Diffusion MRI: The diffusion protocol used a spherically optimized scheme across four b-values (b0: 20; b = 400 s/mm²: 64; b = 1000 s/mm²: 88; b = 2600 s/mm²: 128). Scans were acquired at a resolution of 1.5 × 1.5 mm with 3 mm slice thickness, and imaging parameters of TR/TE = 3800/90 ms. Further details on the diffusion MRI protocol can be found in a previous publication (Bastiani et al. 2018).

#### Developing Human Connectome Project Data: MRI Processing

We used segmentations of the 3DT2 images into 87 anatomical labels, from which the left and right thalamus masks were extracted. The diffusion MRI data were pre-processed using the dHCP diffusion pipeline (Bastiani et al. 2018), including motion correction and eddy current distortion corrections with methods in FSL (Andersson and Sotiropoulos 2016). To further process the dMRI, we applied bias-field correction using MRtrix3 to address intensity inhomogeneity, as recommended for group-wise FOD estimation (Tustison et al. 2010). We then aligned the 3DT2 thalamus masks to diffusion space by extracting the median b0 image from the diffusion data and aligning the 3DT2 images to this median b0 volume using FLIRT in FSL (Jenkinson and Smith 2001) with linear registration (6 DOF). The thalamus masks were then aligned with both linear and non-linear transformations, following the same procedure used for the scan-rescan dataset.

#### Zurich Neonatal Data: Demographics and MRI Acquisition

This dataset included healthy term-born infants, recruited from January 2011 to April 2019 by the Heart and Brain Research Group Zurich at the University Children’s Hospital Zurich. Inclusion criteria was specified birth at > 36 weeks gestation with unremarkable postnatal adaptation. The study was approved by the Ethical Committee of the Canton Zurich, with parental consent provided for all participants.

Brain MRIs were performed on a 3.0T GE Signa MR750 scanner with an 8-channel coil. The anatomical MRI included a T2-weighted fast-spin-echo sequence in axial, coronal, and sagittal planes (TE/TR: 97/5900 ms, flip angle: 90°, slice thickness 2.5 mm, slice gap 0.2 mm). Further imaging details have been reported previously (Bertholdt et al. 2013). Diffusion tensor imaging (DTI) was acquired in the axial plane using a pulsed gradient spin-echo echo-planar imaging sequence (TR/TE 3950/90.5 ms, FOV 18 cm, matrix 128 × 128, slice thickness 3 mm) with 35 diffusion encoding gradient directions (b-value: 700 s/mm^2^) and four b = 0 images. MRI acquisition details for both datasets have been documented in previous work (Feldmann et al. 2020). After DTI quality check, this dataset included 42 infants (19 females, 23 males), birth age ranged from 37.7 to 41.6 (mean ± SD: 39.58 ± 1.22), scan age ranged from 39 to 48.7 (mean ± SD: 42.55 ± 1.96) weeks, respectively.

#### Zurich Neonatal Data: MRI Processing

Axial, coronal and sagittal T2-weighted MR images were reconstructed into a 3DT2 image with an isotropic resolution of 0.5 mm using the SVRTK method (Kuklisova-Murgasova et al. 2012). dMRI data were preprocessed using an in-house pipeline, which included denoising (Veraart et al. 2016), motion and distortion correction (Andersson et al. 2003; Andersson and Sotiropoulos 2016), and bias-field correction (Tustison et al. 2010) using MRtrix3, FSL, ANTs (https://github.com/annspe/connectome_pipeline). Thalamic masks on the 3DT2 images were manually annotated using 3D Slicer (Kikinis et al. 2014). Alignment of the 3DT2 thalamus masks to the diffusion MRI space in this dataset followed the same transformation methodology used for the dHCP data.

### Fiber Orientation Distribution (FOD) Estimation from dMRI

We used local diffusion features to delineate thalamic topography through a data-driven clustering method. Constrained spherical deconvolution (CSD) in MRtrix3 was applied to estimate white matter fiber orientation distributions (WM-FODs) from the dMRI signal in each voxel. The WM-FODs were represented using a spherical harmonics (SH) basis with orders 0, 2, 4, 6, and 8, corresponding to vectors with dimensions 1, 6, 15, 28, and 45, respectively.

**Fig. 1:**
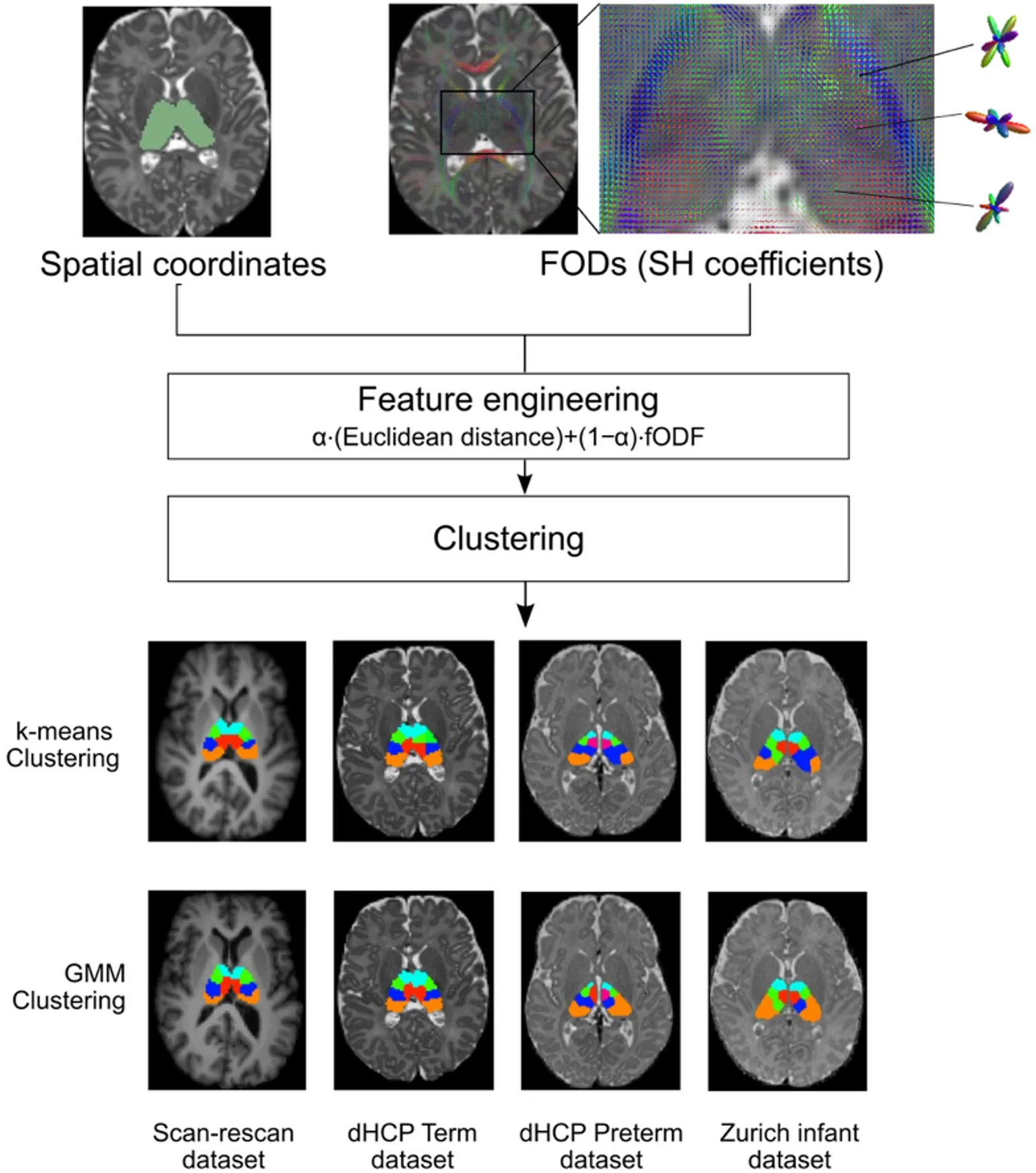
Overview of the clustering framework, clustering results, and WM-FOD glyphs for the same MRI level. dMRI-based segmentation of thalamic nuclei was performed using K-means and GMM clustering algorithm with optimized feature parameters. The images display individual results of thalamic nuclei segmentation and corresponding WM-FOD glyphs for each voxels. The bottom two rows illustrate hard-clustering (K-means) and probabilistic clustering (GMM) overlaid on the structural images for each study group. Dataset A (adults, test-retest), Dataset B (dHCP), Dataset C (healthy term-born infant, Zurich dataset)

These high-dimensional feature vectors capture the complex fiber orientations within each voxel. The SH basis provided an angular characterization of the WM-FODs, as depicted in the glyph figure in Fig. 1. This approach allowed us to use simple distance metrics to assess similarities in diffusion properties across WM-FODs (as shown in the formula in Fig. 1), following the clustering methodology of Wassermann et al. (Wassermann et al. 2008) for similar fiber orientations. We first estimated white matter (WM), grey matter (GM), and cerebrospinal fluid (CSF) response functions for each subject using the *dhollander* algorithm in MRtrix3Tissue (https://3tissue.github.io), a fork of MRtrix3 (Tournier et al. 2004). Since comparing WM-FODs across subjects requires a group-averaged response function, different procedures were followed for each dataset.

For dataset A (healthy adults for technical optimization), we used the SS3T model (https://3tissue.github.io) in MRtrix3, averaging response functions from all 30 subjects. For Dataset B (dHCP), group-averaged response functions were derived by averaging the WM, GM, and CSF response functions from the 20 oldest subjects (43–45 weeks), representing relatively mature white matter. For datasets C, we used the average response functions from dataset C.

Each subject’s diffusion MRI signal was then deconvolved into tissue-specific FODs using the single shell three tissue CSD (SS3T-CSD) method, with the respective group-average response functions. This allowed us to use FODs as features for clustering, aiming to delineate distinct neural tracts based on their orientation and diffusion properties. The resulting tissue-specific FODs (WM-FOD, GM-FOD, CSF-FOD) underwent intensity normalization (Tournier et al. 2019). Given that the differentiation of the thalamic nuclei is most likely based on white matter architecture (e.g. axonal bundles), and WM-FODs might have the highest signal-to-noise ratio, we used the WM-FOD for our feature extraction.

### Features for Clustering

To cluster the thalamus into subdivisions that may correspond to functional subunits or nuclei, optimal feature selection was necessary. Our method utilized both WM-FODs and spatial coordinates derived from diffusion MRI data for voxel clustering, following the considerations of a previous work showing the possible superiority of this approach over using long-range connectivity (Battistella et al. 2017).

For each voxel, we created two feature vectors, one for spatial coordinate (x, y, z) and another for WM-FODs, representing by 45-dimensional spherical harmonic coefficients. These spatial and FOD-based features were scaled and concatenated into a fused feature vector, controlled by two hyperparameters: *α* (weight for spatial feature) and *s* (scale for spherical harmonic coefficients). The combined feature vector for voxel was a fusion of scaled spatial and WM-FOD features.

For consistency across different MRI scans and subjects, spatial coordinates were normalized using two methods. First, spatial coordinates were normalized to the range [-1, 1] while preserving the original aspect ratio of the MRI volume. Second, distances between voxel feature representations were computed in the fused feature space. This approach allowed for clustering of thalamus voxels based on both spatial location and diffusion characteristics.

### Clustering

To optimize the clustering hyperparameters *α* and *s*, evaluate within-subject reproducibility, and compare the results to histological/anatomical subdivisions, we applied both K-means clustering and GMM to Dataset A. Based on cytoarchitectural studies, the thalamus can be grouped into seven main subdivisions or nuclei groups (Morel et al. 1997), and diffusion MRI studies have also supported a cluster number of seven for the thalamus (Battistella et al. 2017). We used the Euclidean distance as the metric. Cluster number was not selected solely from unsupervised validity indices, as this study aimed to examine biological interpretable thalamic subgroups, and therefore, we treated the seven Morel-derived nuclei groups as the primary anatomical reference model. Data-driven indices were used as sensitivity checks rather than as the sole criteria for defining the final parcellation.

We employed two clustering methods: k-means clustering and Gaussian mixture models (GMM), using the *scikit-learn* package (Pedregosa et al. 2011). These methods were selected because they can be applied directly to the fused spatial and WM-FOD feature vectors and provide transparent voxel-wise parcellations. K-means was used as a hard-assignment baseline based on nearest-centroid distance, whereas GMM was used as the main clustering approach because it provides probabilistic assignments and can represent uncertainty near cluster boundaries. This local-feature clustering strategy is suitable for neonatal thalamic parcellation because it avoids reliance on long-range tractography, which may be less reliable in pre-myelinated tissue. For a set of observation $\left\{\mathrm X={\mathrm x}_1,{\mathrm x}_2,...,{\mathrm x}_{\mathrm N}\right\}$, where each is a 48-dimensional vector, a GMM represents the data as a mixture of *K* different Gaussian distributions. The likelihood of our data under this model is given by: 

$$p(x) = \sum_{k=1}^{K} \pi_k \cdot \mathcal{N}(x | \mu_k, \Sigma_k)$$

Where N(x|µ_k,_ ∑k ) is the Gaussian distribution for cluster *k*, with mean *µ*_*k*_ and covariance *∑*_*k*_. The term *π*_*k*_ represent the mixing coefficient for the *k-th* Gaussian (*π*_*k*_ represent the mixing coefficient for cluster *k*), satisfying $0<{\pi}_{k}<1$, and $\sum\limits_{k=1}^{K}{\pi}_{k}=1$ .

For each voxel *i*, the fitted GMM provides posterior responsibilities ${\mathrm r}_{\mathrm{ik}}=\Pr\;\left({\mathrm z}_1=\mathrm k\mid{\mathrm x}_{\mathrm i}\right)$, representing the probability of belonging to cluster *k* given the voxel’s fused spatial and WM-FOD feature vector ${x}_{i}$, with ${\textstyle\sum\limits_{\mathrm k=1}^7}{\mathrm r}_{\mathrm{ik}}=1$. Hard voxel labels were derived by maximum a posteriori (MAP) assignment, $\widehat{{\mathrm z}_{\mathrm i}}=\arg_{\mathrm k}{\mathrm{maxr}}_{\mathrm{ik}}$. Voxel-wise assignment certainty can be quantified by assignment confidence (the maximum posterior probability), the margin between the highest and second-highest posterior probabilities, and normalized posterior entropy, ${\mathrm H}_{\mathrm i}=-{\textstyle\frac{\sum\limits_{\mathrm k=1}^7{\mathrm r}_{\mathrm{ik}}\log{\mathrm r}_{\mathrm{ik}}}{\log\;7}}$, with $0\:log\:0=0$. Higher confidence and larger margins indicate greater certainty under the fitted model, whereas higher entropy indicates greater uncertainty.

### Registration and Atlas Re-mapping

For anatomical guidance, we mapped subdivisions from the Morel histological atlas onto the subject’s anatomical space. We used the publicly available version of the MNI152-aligned, voxelized version of the Morel Atlas of the Human thalamus downloaded from the repository of the ETH Zurich https://www.research-collection.ethz.ch/handle/20.500.11850/669887 (Krauth et al. 2009; Jakab et al. 2012). This atlas was first aligned to a neonatal T2-weighted imaging template from the ALBERTs atlas (Gousias et al. 2012) using a statistical shape model alignment, with the thalamus outlines serving as the shape predictor, based on a previously reported method (Jakab et al. 2012).

Subject-specific registration was done in three-steps. First, an initial affine registration (6 DOF) aligned the neonatal T2 template to each subject’s T2-weighted image using FLS’s FLIRT (Jenkinson and Smith 2001). This was followed by a refined affine registration (9 DOF, FLIRT), incorporating thalamic outlines and restricting Y-axis rotations (± 20 degrees) to preserve anatomical orientation. In this step, thalamus outlines were used as guiding features rather than grayscale images. Finally, a non-linear outline-to-outline registration was performed using ANTs (Avants et al. 2008) on binarized thalamic masks to improve local alignment.

Following registration, Morel atlas labels were transferred to subject space. Each thalamic cluster was assigned the label of the nearest overlapping atlas-defined nucleus using majority-vote rules within intersecting voxels. The spatial alignment between clustered regions and atlas-based nuclei was quantified using the Dice similarity coefficient (DSC) and the 95th percentile Hausdorff distance (HD) as defined by Zou et al. (Zou et al. 2004).

For anatomical label assignment of the clusters, the thalamic nuclei from the Morel atlas were grouped into seven functionally and anatomically coherent clusters based on established structural and functional relationships. Table 1 shows the assignment of nuclei from the Morel atlas into these seven groups: (1) Anterior group, (2) Mediodorsal and Central group, (3) Ventral Anterior group, (4) Ventrolateral and Ventromedial group, (5) Ventral Posterior group, (6) Pulvinar and Geniculate group, and (7) Lateral group. This grouping provided a biologically interpretable framework for evaluating thalamic topography across development and disease conditions. Each nucleus is labeled with its abbreviation, corresponding group number, and group name (Table 1).

**Table 1:** Thalamic nuclei grouping based on the morel atlas

Label ID	Abbreviation (Morel atlas)	Nuclei group Number	Nuclei group name
1	AD	1	Anterior
2	AM	1	Anterior
3	AV	1	Anterior
4	CL	2	Mediodorsal and Central
5	CM	2	Mediodorsal and Central
6	CeM	2	Mediodorsal and Central
7	Hb	2	Mediodorsal and Central
8	LD	7	Lateral
9	LGNmc	6	Pulvinar and Geniculate Nuclei
10	LGNpc	6	Pulvinar and Geniculate Nuclei
11	LP	7	Lateral
12	Li	6	Pulvinar and Geniculate Nuclei
13	MDmc	2	Mediodorsal and Central
14	MDpc	2	Mediodorsal and Central
15	MGN	6	Pulvinar and Geniculate Nuclei
16	MV	2	Mediodorsal and Central
17	Pf	2	Mediodorsal and Central
18	Po	5	Ventral Posterior
19	PuA	6	Pulvinar and Geniculate Nuclei
20	PuI	6	Pulvinar and Geniculate Nuclei
21	PuL	6	Pulvinar and Geniculate Nuclei
22	PuM	6	Pulvinar and Geniculate Nuclei
23	Pv	2	Mediodorsal and Central
24	RN	5	Ventral Posterior
25	SG	6	Pulvinar and Geniculate Nuclei
26	STh	5	Ventral Posterior
27	VAmc	3	Ventral Anterior
28	VApc	3	Ventral Anterior
29	VLa	4	Ventrolateral and Ventromedial
30	VLpd	4	Ventrolateral and Ventromedial
31	VLpv	4	Ventrolateral and Ventromedial
32	VM	4	Ventrolateral and Ventromedial
33	VPI	5	Ventral Posterior
34	VPLa	5	Ventral Posterior
35	VPLp	5	Ventral Posterior
36	VPM	5	Ventral Posterior
37	mtt	5	Ventral Posterior
38	sPf	4	Ventromedial

### Bayesian Hyperparameter Tuning

We addressed the challenge of hyperparameter tuning by maximizing a function *g*: *R*^*2*^ → [0,1] where *g* links the hyperparameters *α* and *s* to the DSC value. To optimize this function, we employed Bayesian optimization with a Gaussian process (GP) as the surrogate model. The GP’s capability to model distributions over functions allows it to treat *g* as a sample from these distributions, enhancing search efficiency and reducing computational expenses.

Optimization Model:

$$\mathrm a^\ast,\mathrm s^\ast=\arg\;\max_{\mathrm a,\mathrm s}\;\mathrm E\;\left[\mathrm g\left(\mathrm a,\mathrm s\right)/\mathrm{data}\right]$$

During the optimization process, each new observation *g* (α, s) refines the GP model, diminishing uncertainty and focusing on promising region of the hyperparameter space. We implemented this approach using the Bayesian-optimization package for Python (Kim and Choi 2023).

### Evaluation of Clustering Accuracy

We assessed the accuracy and reproducibility of both K-means and Gaussian mixture model (GMM) clustering approaches through reproducibility analysis and anatomical validation. Detailed evaluation results for the K-means clustering are provided in the Supplementary Materials. First, to evaluate reproducibility, we use the Dice Similarity Coefficient (DSC) to measure the overlap between clustering results from repeated scans of the same subject. For two scans, *A* and *A’*, and a thalamus subdivision *C*, the DSC for the scans across the 7 subdivisions is defined as:

$$\mathrm{DSC}\left(A,{A}^{{\prime\:}}\right)=\frac{1}{7}\sum\limits_{i=1}^{7}\frac{2\left|{A}_{i}\cap\:{A}_{i}^{{\prime\:}}\right|}{\left|{A}_{i}\right|+\left|{A}_{i}^{{\prime\:}}\right|}$$

Where *A*_*i*_ and *A*_*i*_*’* represent the region of interest for the *i*-th subdivision in scans *A* and *A’*, respectively. We used the DSC implementation from MONAI (Jorge Cardoso et al. 2022). The DSC offers a quantitative measure of spatial overlap between two samples and is computed for individual subjects as the mean DSC across the 7 distinct thalamic functional groups.

Second, to evaluate the anatomical relevance of our clustering method, we compared the clustering results to histological Morel atlas maps, which served as anatomical guidance. We calculated the DSC between the clustering output and the histology-based atlas map and assessed how well the clustering method identified true anatomical or functional regions.

### Statistical Tests

We tested age-related changes in both absolute and relative volumes of data-driven thalamic clusters as a proxy for thalamic topography. Relative volumes were computed by normalizing each cluster volume to the total thalamic volume. Robust linear regression models were used to assess age-related volume changes, implemented via the Robust linear Model (RLM) function in the *statsmodels* Python package (Seabold and Perktold 2010). All regression models used the Huber loss function to reduce sensitivity against outliers. Separate models were fitted for both absolute and relative thalamic volumes, with postmenstrual age at MRI scan as the independent variable.

To account for potential cohort (between-center) effects, we first fit models on the pooled developmental dataset (dHCP and Zurich newborns), including dataset as a covariate. Next, we additionally run subgroup-specific (preterm born dHCP, term born dHCP, Zurich infants) to explore group-level differences in developmental trajectory. To address the issue of potential site and scanner biases, we harmonized the absolute volumes of each cluster using ComBat-GAM (Pomponio et al. 2020), implement via the *neuroCombat* package in Python. Harmonization was done separately for each of the seven clusters. Harmonized volumes were then used to recompute relative volumes, and all regression analyses were repeated.

To evaluate the robustness of the pooled analysis, we tested for the main effect of cohort and cohort-by-age interaction terms. Additionally, we examined sex differences in developmental trajectories by including sex as a covariate. Re-mapping of clusters to the Morel atlas resulted in certain nuclei to be mapped onto the same label, therefore, clusters with zero volume were treated as missing and excluded from cluster-specific analyses. Final sample sizes varied by cluster and are summarized in Table 2.

**Table 2:** Age-related changes of thalamus cluster volumes. Harmonized absolute volume robust regression statistics for preterm dHCP, term dHCP, and Zurich infants

Cohort		Cluster 1	Cluster 2	Cluster 3	Cluster 4	Cluster 5	Cluster 6	Cluster 7
Preterm dHCP	N	34	48	48	48	21	41	44
	p	0.129	0.006	< 0.001	0.022	0.129	0.003	0.070
	pFDR	0.130	0.014	< 0.001**	0.039	0.130	< 0.001	0.098
	Adj. R	0.073	0.104	0.263	0.100	0.102	0.177	0.061
Term dHCP	N	44	68	68	68	32	68	63
	p	0.767	< 0.001	< 0.001	0.023	0.003	< 0.001	0.174
	pFDR	0.0.767	< 0.001**	< 0.001**	0.032	0.005	< 0.001**	0.203
	Adj. R	-0.028	0.126	0.42	0.036	0.207	0.268	0.019
Zurich Neonates	N	33	27	41	41	5	41	41
	p	0.096	0.144	0.085	< 0.001	0.007	0.005	0.963
	pFDR	0.135	0.167	0.135	< 0.001	0.016	0.016	0.963
	Adj. R	0.062	0.073	0.066	0.297	0.046	0.139	-0.026
Pooled Dataset	N	111	143	157	157	58	157	148
	p	< 0.001	< 0.001	< 0.001	< 0.001	< 0.001	< 0.001	< 0.001
	pFDR	< 0.001**	< 0.001**	< 0.001**	< 0.001**	< 0.001**	< 0.001**	< 0.001**
	Adj. R	0.231	0.367	0.611	0.249	0.365	0.523	0.130

FDR-corrected p-values are marked with *p* < 0.05 (*) and *p* < 0.001 (**)

*N *number of cases, *p p*-value, *pFDR *FDR-corrected *p*-value, *Adj. R² *Adjusted R squared

## Results

### Parameter Optimization

#### Grid Search Based on DSC Between K-means Cluster and the Morel Atlas

**Fig. 2:**
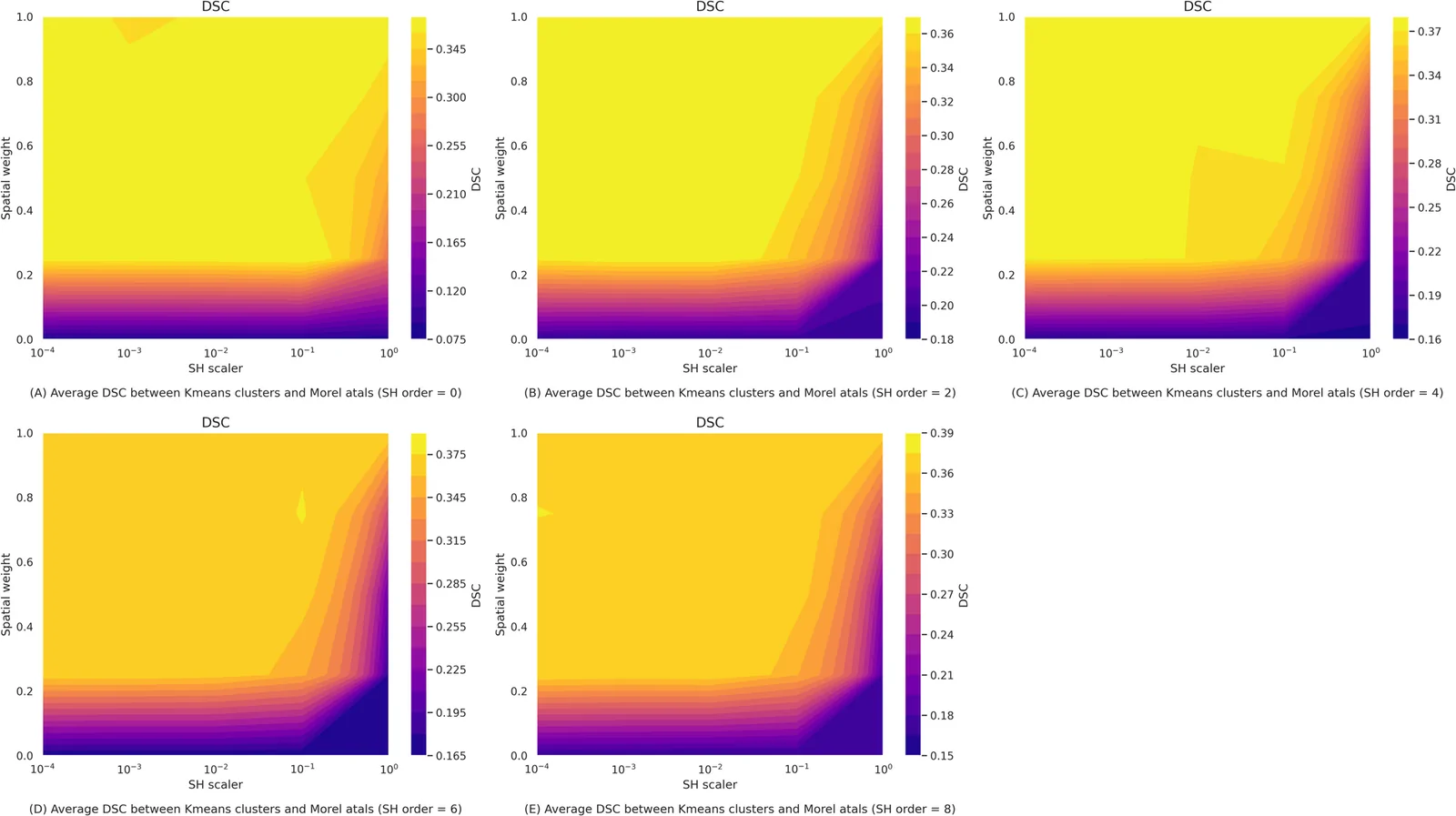
Dice Similarity Coefficient (DSC) between K-means clusters and the Morel atlas across a grid of spatial weights and SH scaling values. Each panel corresponds to a different SH order. **A** SH order=0, **B** SH order=2, **C** SH order=4, **D** SH order=6, **E** SH order=8. Warmer colors indicate higher anatomical alignment (higher DSC). Alignment generally improves with increased spatial weights but also peaks at lower spatial weights when combined with moderate SH scaling (SH orders 4 and 6), highlighting the contribution of diffusion-derived features to anatomical correspondence

To refine clustering performance and evaluate anatomical validity, we optimized spatial weighting and SH scaling parameters using DSC between K-means clusters and the digital Morel atlas. As shown in Fig. 2, high spatial weights generally improved alignment, consistent with trends observed in scan-rescan reproducibility analysis (Supplementary Fig. 2). Lower SH orders (4 and 6) produce consistently higher DSC values across a broader ranges of spatial weightings, while SH order 8 provided no additional benefit.

#### Bayesian Optimization

As a second step in optimizing clustering parameters, we performed Bayesian optimization to refine clustering parameters, using fixed maximum spatial weights of 0.9, 0.8, 0.7, and 0.5. Our results suggest that optimal DSC values for thalamus segmentation can be achieved using spatial weights significantly below 1. As shown in Fig. 2 and Supplementary Table 1, high anatomical alignment requires careful calibration of both spatial weight (*α*) and spherical harmonic scaler (*s*), with moderate SH contributions proving important for thalamus segmentation. Although exact optimal values are dataset-dependent, our results indicate that *α* value near 0.5 and *s* values around 10^− 2^ provided a good balance between spatial proximity and diffusion-derived features.

Further distance-based analysis confirmed that equal contribution of spatial and SH-derived distances produced a stable DSC of approximately 0.7. The top 10 DSC values from Bayesian optimization, with maximum spatial weights of 0.8 (left) and 0.9 (right), are detailed in Supplementary Table 2.

#### Scan-rescan Reproducibility

**Fig. 3:**
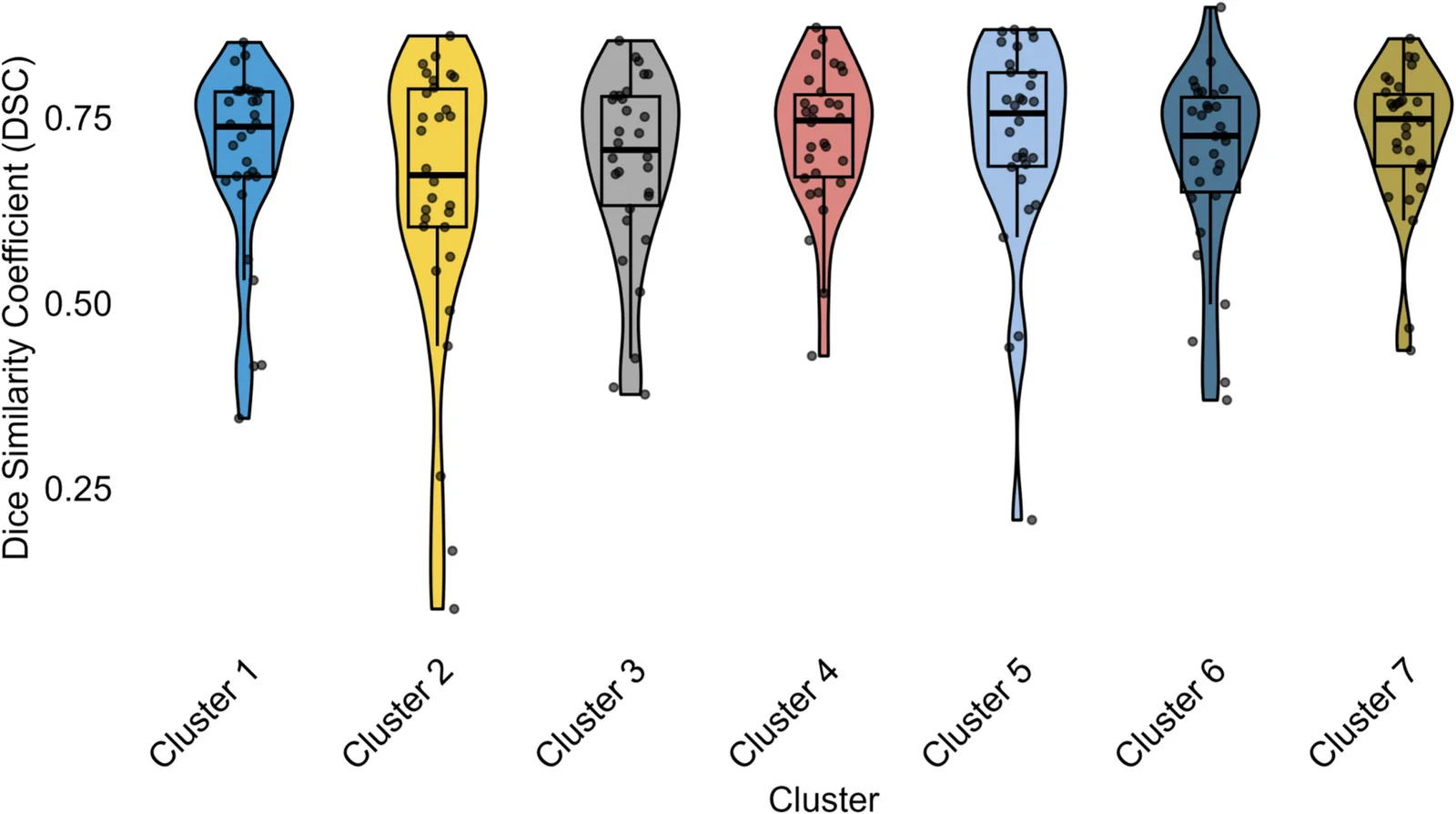
Scan–rescan reproducibility of GMM-derived thalamic clusters. Violin plots show the distributions of Dice similarity coefficients (DSC) for the seven clusters in the scan–rescan cohort. The x-axis denotes cluster identity, and the y-axis denotes DSC. For each voxel, the GMM provides posterior responsibilities, Pr(cluster k | voxel). Hard labels were obtained by maximum a posteriori (MAP; argmax) assignment, selecting the cluster with the highest posterior responsibility, and DSC was calculated from these hard labels. Black solid lines indicate the mean DSC for each cluster. DSC measures scan–rescan overlap; voxel-wise assignment confidence, posterior margin, and normalized posterior entropy are defined in "Clustering" section and are not displayed here

GMM-based clustering showed moderate scan-rescan reproducibility (Fig. 3), with overall lower and more variable Dice scores. The average cluster-wise Dice coefficient for GMM ranged from 0.65 to 0.73, indicating less stable segmentation, with some clusters showed considerable inter-subject variability. For comparison, detailed reproducibility results for K-means clustering are provided in the Supplementary Materials (Supplementary Fig. 3, Supplementary Table 3).

Although the silhouette and gap statistics favored a two-cluster solution, this was interpreted as the strongest coarse-scale data-driven separation rather than the target anatomical parcellation. The final seven-cluster solution was not constrained to follow the borders of the two-cluster solution, but was selected a priori as an atlas-informed, biologically interpretable parcellation based on the Morel nuclei grouping.

### Similarity Between Histology Atlas-based Thalamic Nuclei and the WM-FOD-based Clusters

**Fig. 4:**
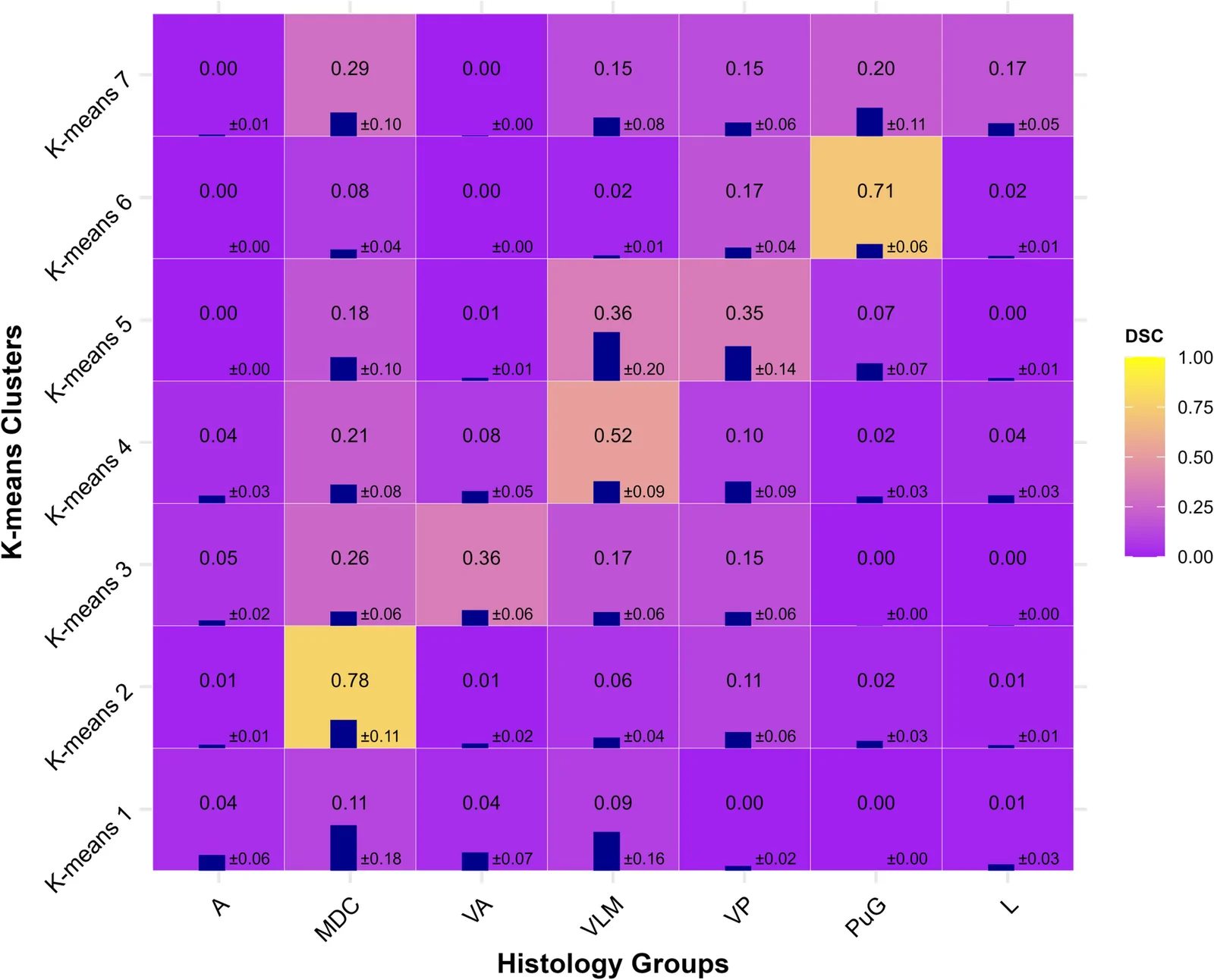
Anatomical correspondence between GMM-based thalamus clusters and atlas-defined thalamic nuclei. The X-axis shows the seven Morel atlas nuclei groups (A, MDC, VA, VLM, VP, PuG, L), and the Y-axis represents GMM clusters. Each cell displays the mean Dice coefficient of volume overlap across 30 subjects (Dataset A), with standard deviations indicated by blue bars. Compared to K-means, GMM clusters show lower and more variable alignment with anatomical nuclei, particularly outside the MDC and PuG regions. Brightness encodes average DSC values (0-1)

Anatomical correspondence between clusters and atlas-defined nuclei was assessed in the scan-rescan dataset (Fig. 4). Cluster 2 showed strong and consistent alignment with the Mediodorsal and Central (MDC) group (mean DSC: 0.78 ± 0.11), followed by Cluster 6 moderately overlap with the Pulvinar and Geniculate (PuG) group (0.71 ± 0.06), and Cluster 4 partially align with the Ventrolateral and Ventromedial (VLM) group (0.52 ± 0.09). Other clusters showed more distributed or minimal overlaps, indicating lower anatomical specificity. Full DSC values for each GMM cluster and corresponding Morel-defined nuclei are provided in Supplementary Table 4. For comparison, K-means-based anatomical alignment results are available in Supplementary Fig. 4 and Supplementary Table 5.

### Characterizing Developmental Trajectories of Thalamic Topography in the Perinatal Period

Table 2 summarized the robust regression results for age-related changes in absolute volume after harmonization. In the preterm dHCP group, significant positive associations with age were observed in Cluster 2 (MDC), 3 (VA), 4 (VLM), and 6 (PuG) (all FDR-corrected *p* < 0.05), with the strongest effect see in Cluster 3(VA) (adjusted *R*^*2*^ = 0.263). Term dHCP infants (*n* = 68), showed significant increases in Clusters 2 (MDC), 3 (VA), 4 (VLM), 5 (VP), and 6 (PuG), again with Cluster 3 (VA) showing the largest age-related growth (adjusted *R²* = 0.42).

In the Zurich cohort (*n* = 41), significant associations were found in Clusters 4 (VLM), 5 (VP), and 6 (PuG), though the effect size was smaller overall (e.g., Cluster 4: adjusted *R²* = 0.297). Cluster 5 (VP) was only present in 5 Zurich newborn subjects. When pooling data from all cohorts (preterm born dHCP, term born dHCP, and Zurich infants), significant growth was seen across all seven clusters (all FDR-corrected *p* < 0.001), with the highest adjusted *R*^*2*^ in Cluster 3 (VA) (adjusted *R*^*2*^ = 0.610), followed by Cluster 6 (PuG) (adjusted *R*^*2*^ = 0.521) and Cluster 2 (MDC) (adjusted *R*^*2*^ = 0.367). These results showed consistent thalamic volume growth during the perinatal period, particularly in MDC, VA, and pulvinar-related regions.

Thalamus topography changes are summarized as the harmonized relative volume, which are detailed in Table 3. Relative volumes were largely stable across cohorts, with only a few significant age-related effects. In the pooled dataset, only Clusters 3 (VA), 6 (PuG), and 7 (L) showed significant associations with age (FDR-corrected *p* < 0.05), though the effect sizes were modest (adjusted *R²* ranging from 0.039 to 0.101). At the individual cohort level, no significant associations were found after FDR correction, except for Cluster 5 (VP) in the Zurich cohort (FDR-corrected *p* < 0.001), but this was based on a very small sample (*n* = 5). These findings suggest that while thalamic nuclei grow in absolute volumes, their relative proportions remain largely preserved during the perinatal period.

**Table 3:** Age-related changes of thalamus topographical cluster volumes. Harmonized relative volume robust regression statistics for preterm dHCP, term dHCP, and Zurich infants

Cohort		Cluster 1	Cluster 2	Cluster 3	Cluster 4	Cluster 5	Cluster 6	Cluster 7
Preterm dHCP	N	34	48	48	48	21	41	44
	p	0.117	0.776	0.398	0.334	0.609	0.130	0.767
	pFDR	0.233	0.776	0.398	0.334	0.959	0.130	0.767
	Adj. R	0.036	-0.022	-0.018	-0.021	-0.036	0.0001	-0.022
Term dHCP	N	44	68	68	68	32	68	63
	p	0.121	0.348	0.283	0.820	0.536	0.894	0.458
	pFDR	0.121	0.348	0.283	0.820	0.933	0.894	0.458
	Adj. R	0.032	-0.006	0.003	-0.014	-0.003	-0.015	-0.011
Zurich Neonates	N	33	27	41	41	5	41	41
	p	0.798	0.363	0.404	0.061	< 0.001	0.984	0.204
	pFDR	0.931	0.565	0.565	0.213	< 0.001	0.984	0.475
	Adj. R	-0.030	-0.015	0.003	0.065	-0.031	-0.027	0.021
Pooled dataset	N	111	143	157	157	58	157	148
	p	0.510	0.999	< 0.001	0.207	0.929	0.003	0.005
	pFDR	0.510	0.999	< 0.001**	0.207	0.992	0.003*	0.012*
	Adj. R	-0.006	-0.016	0.101	0.001	-0.0402	0.039	0.042

FDR-corrected p-values are marked with p < 0.05 (*) and p < 0.001 (**)

*N *number of cases, *p p*-value, *pFDR *FDR-corrected *p*-value, *Adj. R² *Adjusted R squared

To evaluate potential cohort effects, we tested for both group differences and age-by-group interactions in the regression models using harmonized absolute volumes. Across all seven clusters, no significant main effects of cohort (all FDR-corrected *p* > 0.69) and age-by-group interactions (all FDR-corrected *p* > 0.27) were observed, except for a trend-level interaction in Cluster 3 (Ventral Anterior group) in the term-born group (uncorrected *p* = 0.019, FDR-corrected *p* = 0.27). These results suggest that the observed developmental trajectories are consistent across cohorts and not driven by cohort-specific biases. Detailed results are provided in Supplementary Table 6.

Sex effects were tested on harmonized absolute thalamic cluster volumes using robust linear regression, including PMA at scan, gender, and their interaction as predictors. Across all seven clusters, no significant main effect of gender or age-by-gender interactions were observed after FDR correction (all FDR-corrected *p* > 0.13). No gender-related differences in developmental trajectories were detected. These findings suggest that gender does not significantly influence thalamic volumetric development during the perinatal period. Detailed results of the gender effect analysis are provided in Table 4.

**Table 4:** Summary of gender and age-by-gender effects on harmonized thalamic volumes

Cluster	Gender effect (FDR-corrected *p*-value)	Age-Gender interaction (FDR-corrected *p*-value)
Cluster 1	0.911	0.523
Cluster 2	0.678	0.133
Cluster 3	0.600	0.594
Cluster 4	0.310	0.888
Cluster 5	0.595	0.888
Cluster 6	0.958	0.189
Cluster 7	0.533	0.712

## Discussion

**Fig. 5:**
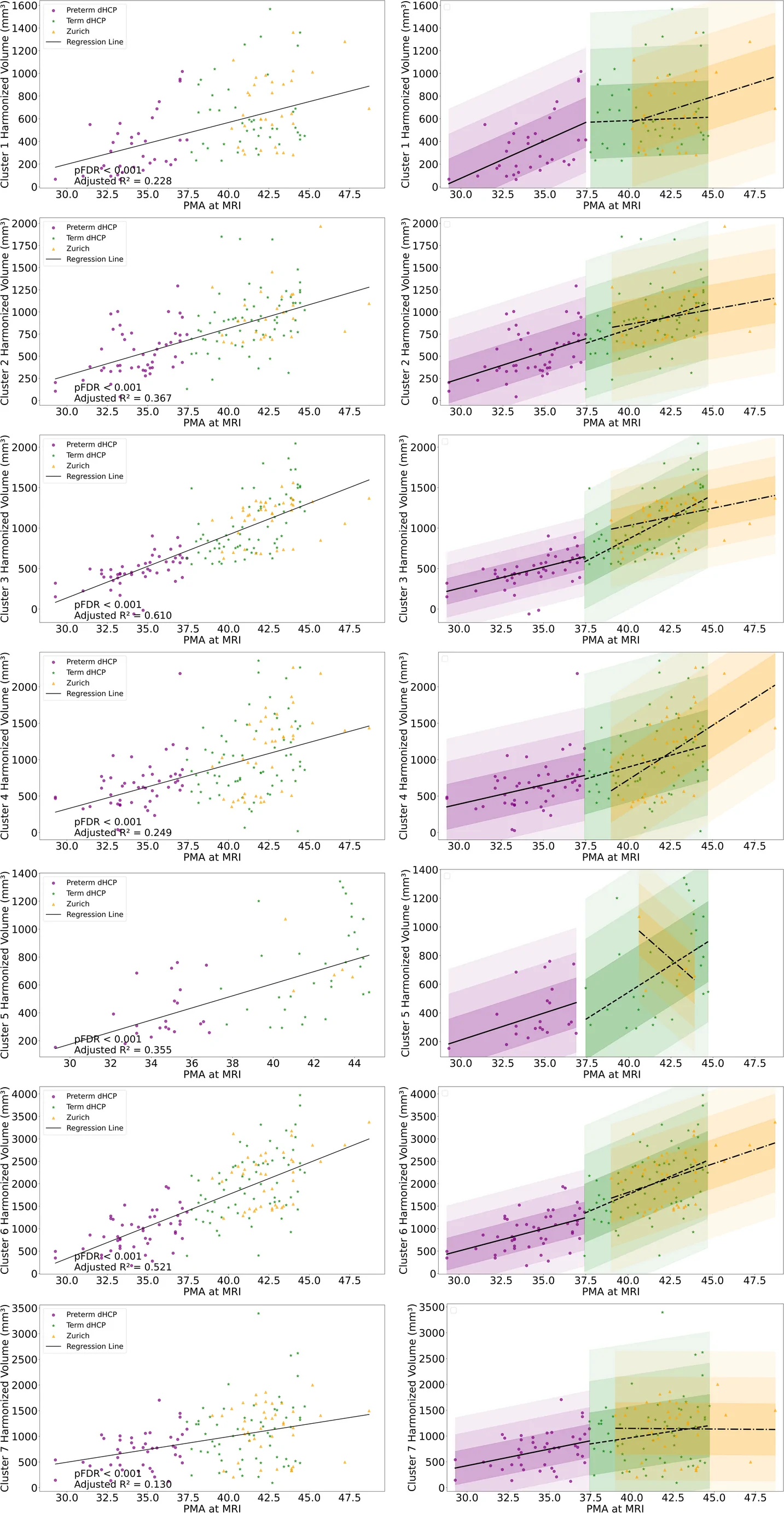
Developmental trajectories of thalamic cluster absolute volumes. The left panel displays the combined regression line across all three cohorts, with dots in distinct colors representing preterm dHCP, term dHCP, and Zurich neonate cohorts. The right panel shows cohort-specific regression lines, with shaded areas indicating variance within ± 1, ±2, and ± 3 standard deviations

The perinatal and early postnatal period is critical in brain development due to the rapid increase of sensory stimuli to the brain, and due to the critically important adaptation of the newborn to its new environment. Our study aimed to use diffusion MRI based parcellation of the thalamus as a proxy to reveal how thalamic structure - topography - is organized in this period. Our study shed light on the developmental dynamics of the thalamus over this developmental period, revealing a positive correlation between absolute thalamic nuclei volumes and scan age as an expected consequence of brain growth (Fig. 5). This is consistent with biological processes such as the maturation of thalamocortical (TC) connection and increased white matter volume as the brain matures, which leads to proportionally increasing thalamus volume (Kostović 2020).

**Fig. 6:**
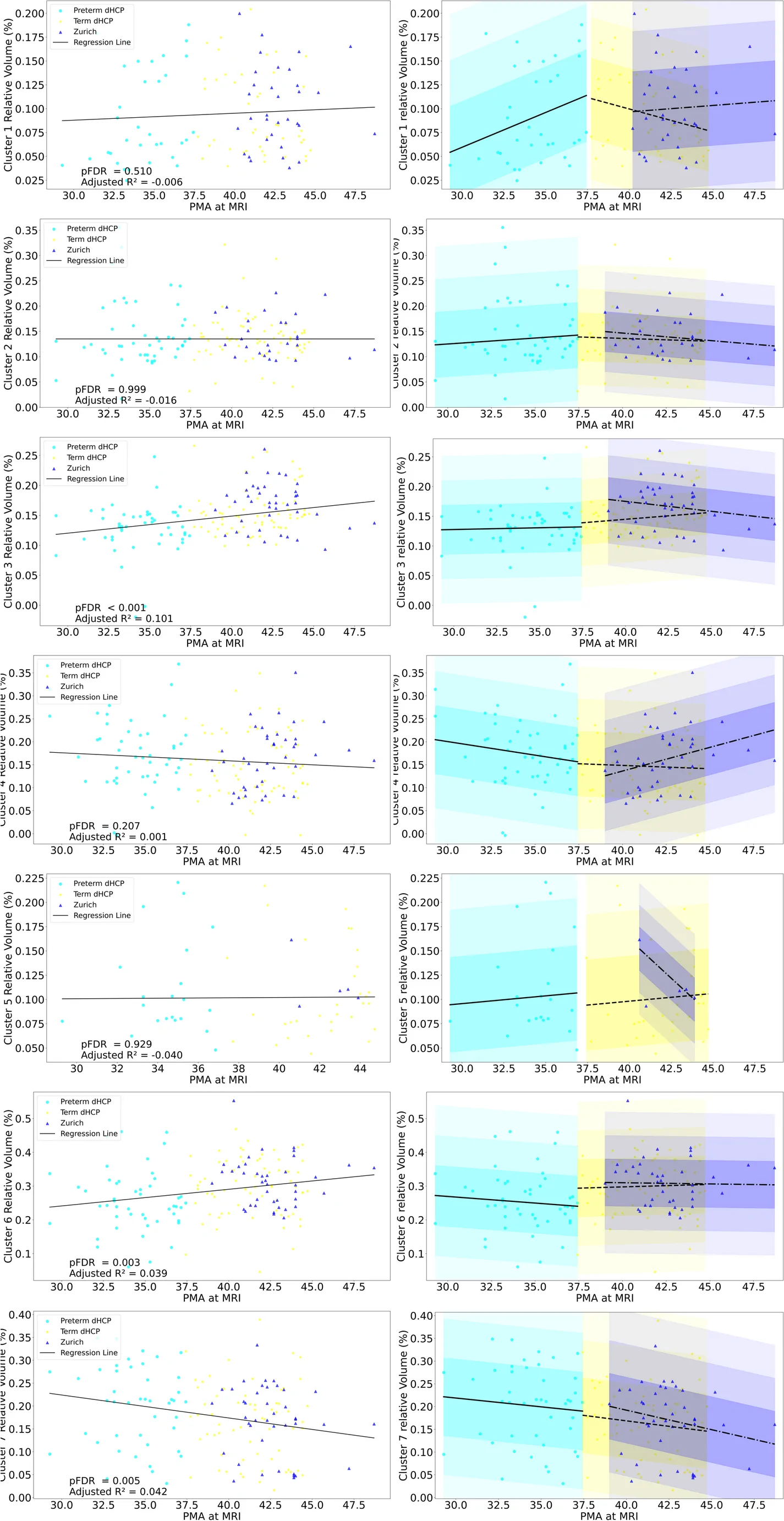
Developmental trajectories of thalamic relative volumes. The left panel presents the pooled regression line across all three cohorts, with dots in three different colors representing preterm dHCP, term dHCP, and Zurich neonate cohorts. The right panel shows cohort-specific regression lines, with shaded areas indicating variance within ± 1, ±2, and ± 3 standard deviations

Thalamus topography, captured by relative volumes, showed a stable pattern (Fig. 6). This stability supports basic research findings suggesting that the topographical organization of the thalamus is established early, following the initial formation of TC circuits, and might remain stable thereafter (Caviness et al. 1980; Molnár et al. 1998; Kostović and Jovanov-Milošević 2006; Kostović and Judaš 2010). This concept aligns with the *handshake hypothesis* (Molnár and Blakemore 1995) and the timeline of thalamocortical connection development, proposing that once these early TC connections are established - around the 18-22nd week of gestation in humans - the overall brain structural connectivity topology remain largely stable. However, our results further suggest that synaptic refinement and ongoing myelination in this period affected the thalamic nuclei homogeneously, not leading to any topological shift in the perinatal and preterm period. Moreover, we did not find that preterm and term-born infants, as well as Zurich neonates, show comparable relative volumes across clusters after harmonization, but this should interpret as an age- and sample-specific observation rather than evidence that prematurity has no effect on thalamic topography organization. In particular, the absence of systematic term-equivalent scans in preterm infants limits direct comparison with age-matched term-born neonates. This is counter-intuitive knowing that thalamocortical connectivity was found to be affected by prematurity (Ball et al. 2012, 2013, 2015). However, our findings reflect topology rather than connectivity strength, and second, our analytical methods including limitations in the clustering process, might not be sensitive enough to capture potential prematurity induced changes, if they are mild.

While the overall topography remained stable across the perinatal period, we observed age-related relative volume changes in specific clusters, such as Cluster 3 (Ventral Anterior group) showed relative volume increase with age. In contrast, Cluster 7 (Lateral groups) showed a relative volume decrease with age. These findings are moderate in statistical significance and therefore might be confounded by clustering uncertainties. However, this finding is consistent with indirect evidence of developmental neuronal exuberance in the MD nucleus (Abitz et al. 2007). The MD nucleus, which has predominantly prefrontal connections and may cluster with the VA nucleus due to its close proximity, could partially explain the observed thalamic changes with gestational age as an indirect sign of selective reorganization of thalamo-frontal connections.

Our study did not statistically evaluate voxel-wise GMM assignment certainty in relation to age or cohort. Therefore, whether assignment confidence, posterior margin, or normalized posterior entropy varies with development or between cohorts cannot be concluded from our data. All volumetric analyses relied on hard maximum-probability labels without weighting by posterior confidence.

Clustering the thalamus based on MRI features offers an emerging approach for delineating its internal architecture, but its biological validity remains disputed. In this study, we evaluated a segmentation framework that uses local diffusion properties and spatial features, building on previous methods that used lower angular resolution data or arbitrary feature weighting methods (Mang et al. 2012; Kumar et al. 2015; Battistella et al. 2017). Consistent with prior studies, we found that anatomical alignment with the Morel atlas (Morel et al. 1997; Krauth et al. 2009) and segmentation reproducibility were similar when using either the primary eigenvector or the full 45-dimensional FODs (Mang et al. 2012; Kumar et al. 2015). However, relying solely on local diffusion features, without spatial information, reduced both anatomical correspondence and scan-rescan reliability. Incorporating moderate-order spherical harmonic (SH) features (orders 4 and 6), together with spatial proximity, improved the biological plausibility of the thalamic segmentation, while high-order SH terms offered limited added value. These findings suggest that biologically meaningful thalamic segmentation can be achieved without very high-angular resolution data, which is potentially applicable to clinically acquired dMRI data, such as that in the Zurich infant cohort in our work. Our framework generalized well across scanners, developmental stages, and in infants born prematurely, supporting its application for studying thalamic maturation and pathology.

Comparisons with the atlas-based labels showed that our diffusion MRI-based thalamic segmentation capture key anatomical subdivisions with good spatial correspondence only in a handful of nuclei groups. In particular, the medial dorsal and central nuclei group, pulvinar, and geniculate group showed substantial overlap with atlas-defined regions. Some clusters, such as Cluster 4 and Cluster 3 showed mixed correspondence, overlapping multiple nuclei. These discrepancies may reflect methodological differences in how labels are acquired. While the Morel atlas delineates nuclei based on cytoarchitectural criteria, our segmentation is driven by local diffusion properties, which likely reveal fiber orientations of thalamic projections. Additionally, we used a distance based assignment of cluster solutions to labels (based on the Hausdorff distance), which may favor larger nuclei, influencing spatial alignment. Despite these factors, the overall correspondence supports the biological validity of diffusion-driven clustering approaches for mapping thalamic subdivisions. Together with reproducibility across different dataset, these findings suggest that dMRI-based segmentation offers a robust and anatomical meaningful framework for studying thalamic development and structure.

In this study, the selection of seven thalamic clusters guided by a combination of biological plausibility and exploratory statistical validation. Anatomically, this choice aligns with prior histological and imaging studies that describe seven major thalamic nuclei groups based on cytoarchitecture, connectivity, and function (Morel et al. 1997; Krauth et al. 2009; Battistella et al. 2017). These nuclei groupings are well-conserved across individuals and provide a meaningful framework for interpreting developmental trajectories and enabling cross-study comparisons. To complement the anatomical rationale, we evaluated statistical support using the elbow method, silhouette score analysis, and gap statistics (Supplementary Method). However, results from these methods were inconsistent, elbow method generally indicated six or seven clusters, whereas silhouette and gap statistics consistently favored a two-cluster solution, likely reflecting a coarse anterior and posterior differentiation. A five-cluster solution, previously reported (Kumar et al. 2015), showed moderate biological correspondence but lacked consistent statistical support in our dataset. Given the inconsistency of purely data-driven statistical metrics across age groups and our aim to maintain biological interpretability, we selected a seven-cluster model as a balanced and robust solution. This is supported by finding that the data-driven cluster optimization contradicted the biologically inspired setting of cluster numbers.

Several limitations should be considered. First, we excluded clusters with zero volume to avoid bias from missing or anatomically absent regions. However, this may introduce selection bias in clusters with higher anatomical variability (e.g., Cluster 5 in the Zurich cohort), potentially reducing statistical power. Second, although combining the dHCP and Zurich cohorts allowed us to better capture the developmental age range, differences in imaging protocols and recruitment may introduce site-related confounds. We addressed this by applying ComBat-GAM harmonization to preserve age-related effects and checked for residual site differences, but we could not run leave-one-cohort-out sensitivity analyses, which limited generalizability. Third, anatomical mapping relied on registration to the Morel atlas, which is based on adult brains. This means that registration inaccuracies and the lack of neonatal-specific atlases mean that some developmental features may not be fully captured. Lastly, the limitation of the absence of a dedicated term-equivalent age preterm cohort. Therefore, developmental age effects and prematurity-related effects cannot be fully disentangled. Adverse effects of preterm birth may emerge only after several days or weeks and would require comparison with age-matched term-born controls at term-equivalent age.

Our main finding of the stability of thalamic volumes across perinatal ages suggests an early establishment of neural connections, consistent with developmental theories, with relatively little impact from ongoing developmental processes like synaptogenesis on thalamic topography.

## Supplementary Information

Supplementary Material 1.

## Data Availability

The study uses open access data (dHCP and scan-rescan MRI data), as well as restricted access patient data from a prospective cohort study. The latter is not available publicly due to patient confidentiality reasons.

## Declarations

We are grateful for the contributions of the "Research Group Heart and Brain", which included the following members who are not listed as authorship contributors: Beatrice Latal, Rabia Liamlahi, Vera Bernet, Hitendu Dave, Oliver Kretschmar, Annette Hackenberg, Achim Schmitz, Markus Weiss, Christian Kellenberger, and Raimund Kottke.
