# Supplementary material for: Stable Thalamic Topography Across Perinatal Development: a Diffusion MRI Study
Source: Brain Topogr. 2026 Oct 12;39(6):104. doi: 10.1007/s10548-026-01257-9 (PMC13658305; doi:10.1007/s10548-026-01257-9)
Supplement: Supplementary file 1 [file 10548_2026_1257_MOESM1_ESM.docx]

**Supplementary Material**

**Supplementary Methods:** Cluster number optimization

For testing the thalamus topography in the developing cohorts, the number of optimal cluster number is unclear. Since there is no single best approach to determine the optimal number of clusters in a dataset, we used three methods to determine the optimal number of clusters for both the k-means and GMM methods. (1) **Elbow method**: This method plots the unexplained variance against the number of clusters. As the number of cluster increases, the explained variance reduces until a point where the benefit diminishes, forming an 'elbow' on the graph. We typically choose the cluster count at this 'elbow' as the optimal number. For each subject, the unexplained variance was calculated for cluster numbers ranging from 2 to 15. This provided insights into the proportion of the dataset's variance that remained unexplained for each clustering solution. A key finding was the identification of a specific cluster number for each subject where the decrease in unexplained variance was no longer substantial, indicating the most suitable number of clusters. (2) **Silhouette Score**: The Silhouette score measures how closely related a thalamus data point is to its cluster compared to other clusters. A higher score suggests well-defined thalamic groupings. For each subject, the optimal number of clusters was determined by identifying the highest average Silhouette score, indicating the clustering configuration where the clusters are most distinct and well-separated.

**(3) Gap statistics:** Gap statistics provide a robust approach to contrast the clustering quality of our observed thalamus data against a baseline of randomly generated reference datasets. This method involves three steps. First, clustering the thalamus data with varying total cluster counts ( k = 1, 2, ..., K), yielding a set of within-dispersion measures for each k. Second, generating B reference datasets. The B reference datasets are generated by Monte Carlo simulation in accordance to the range of each feature from real data. Thus the reference datasets keep the range of the original features of data but without any cluster structure, and calculating with-dispersion measures *W kb* over a range of cluster counts. Third, calculating the Gap statistic, which quantifies the difference between the log of the observed data's dispersion and the expected value from the reference dataset. A larger Gap suggests better clustering for that particular k.

Computed the Gap statistic as:

$$\text{Gap}\left( k \right)=\frac{1}{B}\sum_{b=1}^{B} \log\left( W_{\mathrm{kb}} \right)-\log\left( W_{k} \right)$$

To determine the optimal number of clusters for thalamus topography in developing cohorts, we employed three methods: the Elbow method, the Silhouette score, and Gap statistics. The Elbow method identifies the point where additional clusters yield diminishing returns in explained variance. The Silhouette score measures cluster cohesion and separation, with higher scores indicating better-defined clusters. Gap statistics compare clustering quality against randomly generated reference datasets to identify the most statistically significant clustering solution. Experiment results discussed in the **Supplementary Results.** An illustration of the optimal number clusters selection is shown in **Supplementary Figure 1**

**Supplementary Results:** optimal number of clusters

We employed three statistical methods to search for the optimal number of clusters in our data: the Elbow Method, Silhouette Score Analysis, and Gap Statistics.

In the test-retest dataset (Data A), the elbow method indicated a preference for 6 or 7 clusters to effectively capture the variance in our data, with 18 subjects favoring 7 clusters and 12 subjects suggesting a better fit with 6. The silhouette score identified 2 clusters as optimal across all 30 subjects. Gap statistics confirmed two clusters as the most common optimal solution. While the majority of subjects (18 out of 30) were optimally clustered into 2 groups, 11 subjects showed a preference for 3 clusters, and one subject uniquely favored 5 clusters.

**Supplementary Figure 1**. **Example of a test-retest subject for comparative analysis of optimal clustering approaches.** The left plot shows the Elbow method to a subject from the test-retest data (Dataset A), displaying the mean percentage of unexplained variance against the number of clusters, with a relative clear elbow at seven-cluster solution clusters. The middle plot shows the silhouette analysis for K-means clustering, showing average silhouette scores for different numbers of clusters, with the highest score change observed at the two-cluster solution. The right side plot shows the gap statistic plot, with values across a range of cluster numbers. The optimal number of clusters, indicated by the highest gap value, is two, suggesting that a two-cluster solution provides the most statistically significant differentiation from an expected null reference distribution.

In the dHCP data (Dataset B), the elbow analysis identified six-cluster solution as optimal for explaining data variance in 111 cases, indicating minimal gains with additional clusters. In five instances, seven-cluster solution proved optimal. The silhouette score consistently favored a two-cluster solution as most distinct for our dataset. Gap statistics showed variable optimal cluster numbers. Most subjects were best represented by a two-cluster model, aligning with earlier gestational ages, while a subset, particularly those at later gestational stages (32 weeks and beyond), showed higher optimal cluster numbers. These findings, coupled with analyses of neonatal datasets from Zurich, demonstrate a binary subdivision in earlier developmental stages and increased clustering complexity in later stages.

**Supplementary Methods:** parameters optimization

Grid search based on DSC between scan and rescan

To assess reproducibility, we conducted a grid search across spatial weight and spherical harmonics (SH) scaling using scan-rescan data (Dataset A). As shown in **Figure 2**, high spatial weights consistently yield higher DSC values, indicating the importance of spatial proximity in clustering stability. At lower SH orders (0,2), DSC was primarily influenced by spatial weighting, with minimal effect from SH scaling. In contrast, higher SH orders 4 and 6 showed more stable DSC across SH scalers, suggesting added value from moderate local diffusion features. SH order 6 produced the best balance of reproducibility and parameter stability, while SH order 8 did not yield further improvement, indicating diminishing returns from higher-order SH features.

**Supplementary Results:** reproducibility and anatomical alignment

**Supplementary Figure 2**: **Average Dice similarity coefficient (DSC) across 30 scan-rescan subjects (Dataset A), plotted across a grid of spatial weights and spherical harmonic (SH) scaling values.** Each subplot corresponds to an SH basis orders (0, 2, 4, 6, 8). The X-axis represents the SH scaler (log scale: 10^-4^ to 10^0)^), and the Y-axis denotes spatial weight. Warmer colors indicate higher DSC values, reflecting greater scan-rescan stability. Optimal reproducibility was observed at high spatial weights and intermediate SH orders (4 and 6).

**Supplementary Figure 3: K-means DSC Violin plot of mean DSC values for 30 test-retest subjects across seven clusters.** The X-axis shows K-means clusters (Cluster 1 to Cluster 7), and the Y-axis represents Dice Similarity Coefficient (DSC) values. The width of each violin plot reflects the density of DSC values, with wider sections indicating higher density. The mean DSC for each cluster is marked by a black dashed line. Individual data points (dots) represent each subject’s DSC value from Dataset A, measuring the overlap between scan and re-scan sessions, providing an indicator of clustering reliability.

**Supplementary Figure 4: K-means based clusters of Anatomical correspondence between Kmeans-based thalamus clusters and atlas-defined thalamic nuclei.** The X-axis labels seven atlas-based nuclei: Anterior (A), Mediodorsal and Central (MDC), Ventral Anterior (VA), Ventrolateral and Ventromedial (VLM), Ventral Posterior (VP), Pulvinar and Geniculate (PuG), and Lateral (L). The Y-axis shows the seven K-means clusters. Each cell indicates the mean volume overlap across 30 subjects (Dataset A), with blue error bars representing standard deviation. Brightness corresponds to the mean overlap percentage, reflecting the distribution and variability across subjects (blue code: matrix diagonal, i.e., self-correlation).

**Supplementary Table 1**. Bayesian optimization of 100 feature parameters based on average DSC across 30 subjects.

| DSCs | Log SH coefficient | Spatial weight | DSCs | Log SH coefficient | Spatial weight |
| --- | --- | --- | --- | --- | --- |
| 0.813767733 | -2.933755026 | 0.432840102 | 0.804132132 | -2.808789318 | 0.653510631 |
| 0.813739369 | -2.999547218 | 0.361028328 | 0.804078573 | -1.652061189 | 0.640604768 |
| 0.812198283 | -2.59344781 | 0.221454732 | 0.804034456 | -2.694937791 | 0.168812718 |
| 0.811852681 | -1.868079676 | 0.72162609 | 0.803858106 | -2.703007809 | 0.705479152 |
| 0.811444767 | -4 | 0.553048758 | 0.803747031 | -2.632953376 | 0.517016823 |
| 0.810940296 | -2.755852594 | 0.364549219 | 0.803745141 | -2.460251182 | 0.898054584 |
| 0.810725677 | -2.13162033 | 0.366334117 | 0.803457383 | -1.695036539 | 0.728773066 |
| 0.810724613 | -1.325504883 | 0.9 | 0.803453938 | -2.719425177 | 0.456666089 |
| 0.810490326 | -2.663600215 | 0.303684051 | 0.803349549 | -2.756645682 | 0.072429666 |
| 0.810271065 | -1.779546047 | 0.727087283 | 0.803271955 | -2.596770524 | 0.635011321 |
| 0.809702453 | -2.773317387 | 0.259979974 | 0.802925388 | -2.88865815 | 0.557505752 |
| 0.808953366 | -3.287341985 | 0.0711538 | 0.802825897 | -1.692787509 | 0.439181906 |
| 0.808911237 | -2.824868345 | 0.466120947 | 0.802816477 | -2.479045015 | 0.216547358 |
| 0.808801378 | -2.47739538 | 0.801943376 | 0.802536791 | -2.884420567 | 0.778098794 |
| 0.808733508 | -2.333823595 | 0.611501644 | 0.801846422 | -1.914889976 | 0.648292044 |
| 0.808654306 | -2.663740311 | 0.028294045 | 0.801699318 | -2.376035956 | 0.73125607 |
| 0.80847501 | -2.54395423 | 0.313307911 | 0.801633161 | -1.63467291 | 0.527106605 |
| 0.808019933 | -3.101796234 | 0.369580578 | 0.801454004 | -3.018238555 | 0.557732949 |
| 0.807910795 | -3.171700065 | 0.292969145 | 0.801083557 | -3.331870876 | 0.756855737 |
| 0.807740975 | -2.464155901 | 0.106863173 | 0.8003689 | -2.930060192 | 0.272920096 |
| 0.807671799 | -3.264339578 | 0.089916263 | 0.799997757 | -3.008287472 | 0.898935466 |
| 0.807671796 | -2.603186193 | 0.391997833 | 0.799899434 | -1.780928136 | 0.888397059 |
| 0.80766989 | -2.478759267 | 0.700981874 | 0.799819889 | -2.032013567 | 0.059278978 |
| 0.807586171 | -3.268755323 | 0.085552129 | 0.799617001 | -1.037266736 | 0.782373281 |
| 0.807547218 | -3.018152621 | 0.173842833 | 0.799337745 | -2.283609786 | 0.575355864 |
| 0.807493538 | -2.586649975 | 0.748187853 | 0.799279737 | -1.869075067 | 0.575542548 |
| 0.807457924 | -3.061262182 | 0.272891411 | 0.799142931 | -1.772629912 | 0.479455605 |
| 0.807361315 | -1.830249138 | 0.798493761 | 0.799034317 | -3.175588475 | 0.454974269 |
| 0.80715097 | -2.555835036 | 0.85507682 | 0.799018486 | -2.516395784 | 0.451008525 |
| 0.807146971 | -1.745396389 | 0.804428033 | 0.798803732 | -3.970927982 | 0.9 |
| 0.807026799 | -1.876537335 | 0.886833087 | 0.798571403 | -1.826859255 | 0.650532559 |
| 0.806996509 | -2.87129883 | 0.361710554 | 0.798551902 | -2.607831717 | 0.108289108 |
| 0.806728764 | -1.917219125 | 0.785432944 | 0.798287181 | -2.537135744 | 0.898027483 |
| 0.806480253 | -1.911002711 | 0.64842533 | 0.797556383 | -1.079714737 | 0.778816298 |
| 0.806173634 | -3.266220546 | 0.083104735 | 0.797299147 | -2.902581953 | 0.749543616 |
| 0.806149808 | -2.544693749 | 0.010114717 | 0.797169277 | -2.492877053 | 0.574587092 |
| 0.806048858 | -3.030828327 | 0.450055669 | 0.797150275 | -3.679423062 | 0.702877908 |
| 0.805775372 | -2.365045324 | 0.156992919 | 0.796687121 | -1.278883226 | 0.577669758 |
| 0.805686649 | -1.718362474 | 0.563652533 | 0.795243599 | -1.050605963 | 0.726504873 |
| 0.805623435 | -2.38987602 | 0.038964841 | 0.795126838 | -3.253826836 | 0.233492925 |
| 0.805580572 | -1.727204431 | 0.659391173 | 0.793803143 | -1.03675647 | 0.782873815 |
| 0.805499162 | -2.755737504 | 0.562435735 | 0.746586572 | -0.629683174 | 0.867355964 |
| 0.80544722 | -3.281103621 | 0.100151108 | 0.72646716 | -1.188853455 | 0.295147028 |
| 0.805426441 | -2.929032792 | 0.641444626 | 0.714510045 | -1.666693632 | 0.040724334 |
| 0.805364706 | -2.828185158 | 0.162799967 | 0.655111379 | -0.943168955 | 0.446473765 |
| 0.805327602 | -3.142819853 | 0.18421609 | 0.412450827 | 0.144682915 | 0.886519837 |
| 0.805274074 | -2.661145723 | 0.81628902 | 0.389593069 | -0.164234397 | 0.605842514 |
| 0.805241746 | -1.795280872 | 0.397468123 | 0.30656265 | -0.117394977 | 0.176621886 |
| 0.805203456 | -1.87064267 | 0.46689605 | 0.302968278 | 0.438485155 | 0.402480225 |
| 0.805154152 | -2.792509019 | 0.77128795 | 0.301453952 | -3.799035828 | 0 |
| 0.805110872 | -2.392919646 | 0.517685625 |  |  |  |
| 0.805034995 | -3.999428126 | 0.272099315 |  |  |  |
| 0.80420724 | -3.257775776 | 0.366015314 |  |  |  |

**Supplementary Table 2.** top 10 hyper-parameter combinations by setting the maximum spatial weight as 0.8,0.9 separately

| **DSC** | **Log SH coefficient** | **Spatial weight** | **DSC** | **Log SH coefficient** | **Spatial weight** |
| --- | --- | --- | --- | --- | --- |
| 0.8137 | -2.515 | 0.8 | 0.8137 | -2.934 | 0.432 |
| 0.8130 | -3.616 | 0.295 | 0.8137 | -2.999 | 0.361 |
| 0.8129 | -3.948 | 0.292 | 0.8122 | -2.593 | 0.221 |
| 0.8118 | -3.496 | 0.458 | 0.8119 | -1.868 | 0.722 |
| 0.8118 | -3.455 | 0.003 | 0.8114 | -4.0 | 0.553 |
| 0.8114 | -3.717 | 0.166 | 0.8109 | -2.755 | 0.365 |
| 0.8112 | -4 | 0.8 | 0.8107 | -2.132 | 0.366 |
| 0.8110 | -4 | 0.499 | 0.8107 | -1.326 | 0.9 |
| 0.8108 | -2.884 | 0.692 | 0.8105 | -2.664 | 0.304 |
| 0.8105 | -3.589 | 0.621 | 0.8103 | -1.779 | 0.727 |

**Supplementary Table 3**. K-means clustering Dice coefficient of test-retest/scan-rescan data for 30 subjects.

| Subject | Cluster 1 | Cluster 2 | Cluster 3 | Cluster 4 | Cluster 5 | Cluster 6 | Cluster 7 | Average DSC |
| --- | --- | --- | --- | --- | --- | --- | --- | --- |
| 1 | 0.7917 | 0.8868 | 0.6973 | 0.7946 | 0.8214 | 0.7109 | 0.8315 | 0.804074347 |
| 2 | 0.739 | 0.6861 | 0.7379 | 0.7519 | 0.724 | 0.7766 | 0.797 | 0.755486429 |
| 3 | 0.8471 | 0.8185 | 0.8746 | 0.8256 | 0.722 | 0.6667 | 0.7647 | 0.815082699 |
| 5 | 0.8683 | 0.8123 | 0.8571 | 0.886 | 0.8916 | 0.8247 | 0.8789 | 0.802522272 |
| 6 | 0.885 | 0.8951 | 0.8512 | 0.8523 | 0.8313 | 0.8544 | 0.8218 | 0.825935513 |
| 7 | 0.8239 | 0.893 | 0.8389 | 0.7231 | 0.8613 | 0.7649 | 0.8819 | 0.837963492 |
| 8 | 0.8717 | 0.798 | 0.8341 | 0.8337 | 0.8305 | 0.8081 | 0.8878 | 0.856939495 |
| 9 | 0.7654 | 0.6611 | 0.6641 | 0.8782 | 0.8044 | 0.7766 | 0.9058 | 0.827161819 |
| 10 | 0.8763 | 0.8479 | 0.7525 | 0.7759 | 0.7989 | 0.8647 | 0.7848 | 0.796693683 |
| 11 | 0.7854 | 0.7803 | 0.8429 | 0.8548 | 0.8175 | 0.8788 | 0.8057 | 0.733297229 |
| 12 | 0.8372 | 0.876 | 0.8354 | 0.8 | 0.6824 | 0.8328 | 0.8111 | 0.808641315 |
| 13 | 0.8675 | 0.8606 | 0.8537 | 0.8084 | 0.7918 | 0.8549 | 0.8688 | 0.82844606 |
| 14 | 0.7437 | 0.7749 | 0.7481 | 0.7546 | 0.8026 | 0.7241 | 0.7829 | 0.795444578 |
| 15 | 0.9037 | 0.8446 | 0.8876 | 0.8108 | 0.8454 | 0.8444 | 0.7697 | 0.845258832 |
| 16 | 0.8328 | 0.79 | 0.8477 | 0.8389 | 0.726 | 0.8927 | 0.8696 | 0.820751637 |
| 17 | 0.8131 | 0.8963 | 0.7416 | 0.8098 | 0.7857 | 0.811 | 0.8743 | 0.804950535 |
| 18 | 0.8012 | 0.8418 | 0.8262 | 0.8438 | 0.7966 | 0.8056 | 0.8344 | 0.771282047 |
| 19 | 0.926 | 0.8235 | 0.858 | 0.7774 | 0.7889 | 0.8462 | 0.8551 | 0.848283529 |
| 20 | 0.828 | 0.8269 | 0.8228 | 0.8323 | 0.8362 | 0.7944 | 0.8448 | 0.813670695 |
| 21 | 0.8018 | 0.7799 | 0.8049 | 0.8878 | 0.8497 | 0.7437 | 0.7872 | 0.806338996 |
| 22 | 0.8262 | 0.88 | 0.798 | 0.8103 | 0.8081 | 0.8529 | 0.8213 | 0.828734308 |
| 23 | 0.8276 | 0.7851 | 0.6477 | 0.7338 | 0.8356 | 0.5893 | 0.628 | 0.775160253 |
| 24 | 0.852 | 0.877 | 0.8047 | 0.8025 | 0.885 | 0.8599 | 0.8421 | 0.829637825 |
| 25 | 0.8376 | 0.8867 | 0.7864 | 0.8408 | 0.8822 | 0.8226 | 0.8385 | 0.830929816 |
| 26 | 0.8622 | 0.8393 | 0.8928 | 0.8994 | 0.887 | 0.875 | 0.8877 | 0.837834448 |
| 27 | 0.7535 | 0.8733 | 0.8357 | 0.8341 | 0.8318 | 0.8978 | 0.8125 | 0.812636226 |
| 30 | 0.7609 | 0.7969 | 0.7097 | 0.7931 | 0.7985 | 0.7213 | 0.7261 | 0.766376615 |
| 31 | 0.8084 | 0.7692 | 0.671 | 0.789 | 0.734 | 0.6545 | 0.7788 | 0.787196428 |
| 33 | 0.7422 | 0.812 | 0.7676 | 0.8373 | 0.8991 | 0.7348 | 0.8551 | 0.819454879 |
| 34 | 0.7778 | 0.875 | 0.8621 | 0.8866 | 0.8636 | 0.8462 | 0.8759 | 0.84613353 |

**Supplementary Table 4.** GMM clusters Dice coefficient overlap (mean (SD) with the atlas-based thalamus nuclei groups over 30 subjects.

| GMM/Histology | A | MDC | VA | VLM | VP | PuG | L |
| --- | --- | --- | --- | --- | --- | --- | --- |
| GMM cluster 1 | 0.0399 (0.0636) | 0.1148 (0.1836) | 0.0359 (0.0733) | 0.0933 (0.1568) | 0.0050 (0.0184) | 0.0000 (0.0000) | 0.0111 (0.0260) |
| GMM cluster 2 | 0.0093 (0.0143) | 0.7789 (0.1147) | 0.0069 (0.0187) | 0.0584 (0.0429) | 0.1058 (0.0649) | 0.0249 (0.0299) | 0.0143 (0.0125) |
| GMM cluster 3 | 0.0537 (0.0217) | 0.2587 (0.0576) | 0.3623 (0.0633) | 0.1727 (0.0555) | 0.1511 (0.0562) | 0.0003 (0.0011) | 0.0010 (0.0016) |
| GMM cluster 4 | 0.0373 (0.0320) | 0.2132 (0.0758) | 0.0756 (0.0505) | 0.5193 (0.0901) | 0.1010 (0.0890) | 0.0161 (0.0281) | 0.0374 (0.0330) |
| GMM cluster 5 | 0.0000 (0.0000) | 0.1770 (0.0964) | 0.0061 (0.0127) | 0.3612 (0.1981) | 0.3532 (0.1414) | 0.0657 (0.0713) | 0.0033 (0.0116) |
| GMM cluster 6 | 0.0000 (0.0000) | 0.0818 (0.0370) | 0.0000 (0.0000) | 0.0159 (0.0134) | 0.1733 (0.0447) | 0.7126 (0.0590) | 0.0163 (0.0115) |
| GMM cluster 7 | 0.0036 (0.0060) | 0.2927 (0.0960) | 0.0007 (0.0025) | 0.1464 (0.0762) | 0.1454 (0.0553) | 0.2029 (0.1149) | 0.1745 (0.0522) |

**Supplementary Table 5.** K-means clusters Dice coefficient overlap (mean (SD) with the atlas-based thalamus nuclei groups over 30 subjects.

| K-means/atlas nuclei | A | MDC | VA | VLM | VP | PuG | L |
| --- | --- | --- | --- | --- | --- | --- | --- |
| K-means 1 | 3.19 (5.43) | 9.47 (16.35) | 2.34 (4.98) | 10.47 (17.61) | 0.12 (0.37) | 0.00 (0.00) | 1.08 (2.29) |
| K-means 2 | 0.59 (1.05) | 79.76 (10.62) | 0.15 (0.38) | 6.81 (5.18) | 9.51 (6.26) | 1.42 (1.70) | 1.63 (2.01) |
| K-means 3 | 4.64 (2.19) | 27.89 (6.28) | 34.23 (5.28) | 15.75 (5.27) | 17.33 (5.72) | 0.03 (0.12) | 0.11 (0.22) |
| K-means 4 | 4.04 (2.62) | 19.49 (9.07) | 8.17 (5.47) | 50.12 (9.72) | 14.80 (11.94) | 1.27 (1.42) | 2.10 (2.58) |
| K-means 5 | 0.00 (0.00) | 11.84 (10.66) | 0.48 (1.53) | 35.37 (23.80) | 32.86 (19.45) | 2.79 (3.57) | 0.00 (0.00) |
| K-means 6 | 0.00 (0.00) | 11.67 (3.98) | 0.00 (0.00) | 0.91 (1.07) | 15.29 (4.86) | 70.49 (5.79) | 1.62 (1.13) |
| K-means 7 | 0.20 (0.38) | 26.22 (9.88) | 0.08 (0.28) | 14.60 (8.85) | 17.80 (6.96) | 19.12 (11.18) | 18.62 (4.92) |

**Supplementary Table 6.** Summary of cohort-specific effects and cohort-by-age interaction analysis.

| **Cluster** | **Comparison** | **Effect Type** | **FDR-corrected p-value** | **Adjusted R2** |
| --- | --- | --- | --- | --- |
| Cluster 1 | Term vs Preterm | Group | 0.9688 | 0.2188 |
| Cluster 1 | Zurich vs Preterm | Group | 0.9688 | 0.2188 |
| Cluster 1 | Term — Age | Interaction | 0.5946 | 0.2218 |
| Cluster 1 | Zurich — Age | Interaction | 0.8994 | 0.2218 |
| Cluster 2 | Term vs Preterm | Group | 0.9688 | 0.3605 |
| Cluster 2 | Zurich vs Preterm | Group | 0.9688 | 0.3605 |
| Cluster 2 | Term — Age | Interaction | 0.9607 | 0.3524 |
| Cluster 2 | Zurich — Age | Interaction | 0.8994 | 0.3524 |
| Cluster 3 | Term vs Preterm | Group | 0.9688 | 0.606 |
| Cluster 3 | Zurich vs Preterm | Group | 0.9688 | 0.606 |
| Cluster 3 | Term — Age | Interaction | 0.2726 | 0.6238 |
| Cluster 3 | Zurich — Age | Interaction | 0.8994 | 0.6238 |
| Cluster 4 | Term vs Preterm | Group | 0.6953 | 0.2582 |
| Cluster 4 | Zurich vs Preterm | Group | 0.6953 | 0.2582 |
| Cluster 4 | Term — Age | Interaction | 0.9607 | 0.268 |
| Cluster 4 | Zurich — Age | Interaction | 0.5946 | 0.268 |
| Cluster 5 | Term vs Preterm | Group | 0.9688 | 0.3448 |
| Cluster 5 | Zurich vs Preterm | Group | 0.9688 | 0.3448 |
| Cluster 5 | Term — Age | Interaction | 0.809 | 0.3532 |
| Cluster 5 | Zurich — Age | Interaction | 0.6379 | 0.3532 |
| Cluster 6 | Term vs Preterm | Group | 0.9688 | 0.5156 |
| Cluster 6 | Zurich vs Preterm | Group | 0.9688 | 0.5156 |
| Cluster 6 | Term — Age | Interaction | 0.6379 | 0.5144 |
| Cluster 6 | Zurich — Age | Interaction | 0.8994 | 0.5144 |
| Cluster 7 | Term vs Preterm | Group | 0.9688 | 0.1184 |
| Cluster 7 | Zurich vs Preterm | Group | 0.9913 | 0.1184 |
| Cluster 7 | Term — Age | Interaction | 0.9607 | 0.1161 |
| Cluster 7 | Zurich — Age | Interaction | 0.6379 | 0.1161 |
